# Assembly of Bacterial Genome Sequences from Metagenomes of Spacecraft Assembly Cleanrooms

**DOI:** 10.1128/MRA.01439-20

**Published:** 2021-02-18

**Authors:** Velislava Ilieva, Bruce Steel, Jennifer Pratscher, Karen Olsson-Francis, Michael C. Macey

**Affiliations:** a Faculty of Biology, Medicine and Health, School of Biology Sciences, University of Manchester, Manchester, United Kingdom; b The Lyell Centre, School of Energy, Geoscience, Infrastructure and Society, Heriot-Watt University, Edinburgh, United Kingdom; c AstrobiologyOU, School of Environment, Earth and Ecosystem Sciences, Faculty of Science, Technology, Engineering and Mathematics, The Open University, Milton Keynes, United Kingdom; University of Southern California

## Abstract

Characterizing the microbiome of spacecraft assembly cleanrooms is important for planetary protection. We report two bacterial metagenome-assembled genomes (MAGs) reconstructed from metagenomes produced from cleanroom samples from the Kennedy Space Center’s Payload Hazardous Servicing Facility (KSC-PHSF) during the handling of the Phoenix spacecraft. Characterization of these MAGs will enable identification of the strategies underpinning their survival.

## Abstract (precis)

Characterizing the microbiome of spacecraft assembly cleanrooms is important for planetary protection. We report two bacterial metagenome-assembled genome sequences (MAGs) reconstructed from metagenomes produced from cleanroom samples from the Kennedy Space Center’s Payload Hazardous Servicing Facility (KSC-PHSF) during the handling of the Phoenix spacecraft. Characterization of these MAGs will enable identification of the strategies underpinning their survival.

## ANNOUNCEMENT

To avoid microbial contamination during planetary missions, there are standards for spacecraft bioburden that are maintained by the Committee on Space Research (COSPAR) Planetary Protection Panel (https://cosparhq.cnes.fr/scientific-structure/panels/panel-on-planetary-protection-ppp/). Regulations require that spacecraft be assembled in cleanrooms with defined sterilization protocols (1). Studies characterizing these environments have identified a persistent microbiome (1–5). One such study characterized the microbiome of the Kennedy Space Center’s Payload Hazardous Servicing Facility (KSC-PHSF) during the assembly of the Phoenix spacecraft (1).

In that study by Bashir et al. (1), DNA samples were collected with a biological sampling kit (BiSKits, QuickSilver Analytics) from 1 m^2^ of the floor of the KSC-PHSF during (8 samples) and after (10 samples) the spacecraft assembly (1). DNA extraction was performed using bead beating and an automated DNA extraction instrument (Autolyser A-2 DNA automated platform, Axcyte Genomics). Samples from each sampling period were pooled following extraction. Each sample was amplified using multiple displacement amplification with a REPLI-g single-cell whole-genome amplification kit (Qiagen). DNA was sheared using an E210 instrument (Covaris, Woburn, MA) and then end repaired, A tailed, and ligated to Illumina adaptors according to standard Illumina (San Diego, CA) paired-end (PE) protocols. Metagenomic sequencing of the materials was performed on a HiSeq 2500 sequencing instrument (Illumina) with paired-end 2 × 250-bp read lengths (1). This produced 4,654,014 and 22,355,430 raw reads for the metagenomes collected during spacecraft assembly and after spacecraft assembly, respectively. In this study, default parameters were used for all software. The raw reads were downloaded from NCBI GenBank, and the short and poor-quality sequences were excluded using Trimmomatic v0.39 (6). The reads were assembled into contigs using MEGAHIT v1.1.3 (7) (after spacecraft assembly: 114,901 contigs; *N*_50_, 401 bp; during spacecraft assembly: 12,199 contigs; *N*_50_, 1,130 bp). Contigs were binned into metagenome-assembled genomes (MAGs) using MaxBin v2.2.7 (8). The taxonomic classification, completeness, and contamination of these MAGs were assessed using CheckM v1.1.2 (9). A medium-quality MAG was produced from the metagenome collected during spacecraft assembly (MAG-P1) and a high-quality MAG (MAG-P2) from the metagenome collected after spacecraft assembly (10). The contamination scores for MAG-P1 were improved using VizBin v1.0.0 (11). tRNAs and rRNA genes were identified using Aragorn v1.2.38 (12) and RNAmmer v1.2 (13).

The high-quality MAG was classified as *Rhizobium* (MAG-P2), with completeness and contamination scores of 96.33% and 4.69%, respectively, and 438-fold coverage. MAG-P2 is composed of 468 contigs and contains 1 16S rRNA gene copy, 64 tRNAs, and 7,771 coding sequences (CDSs). The genome size is 8.36 Mb, with 63.1% GC content. CheckM classified MAG-P1 in the genus Acinetobacter, with completeness and contamination scores of 52.90% and 8.62%, respectively, and 863-fold coverage. MAG-P1 is 4.34 Mb, with 39.8% GC content, 1,793 contigs, and 4,253 CDSs.

Members of *Rhizobiaceae* and Acinetobacter were previously detected in the KSC-PHSF, as well as other cleanroom environments (1, 5, 14–20). Further analysis of these MAGs will provide insight into the strategies underpinning survival in cleanroom environments and inform future sterilization strategies.

### Data availability.

The sequences analyzed in this study are available from the NCBI Sequence Read Archive under accession numbers SRX1896153 and SRX1896154. The whole-genome shotgun projects were deposited at DDBJ/ENA/GenBank under accession numbers JAEPRK000000000 (Acinetobacter sp. strain MAG-P1) and JAEPRL000000000 (*Rhizobium* sp. strain MAG-P2). The versions described in this paper are the first versions.
